# Interpersonal violence and suicidality among former child soldiers and war-exposed civilian children in Nepal

**DOI:** 10.1017/gmh.2017.31

**Published:** 2018-02-22

**Authors:** Anvita Bhardwaj, Christine Bourey, Sauharda Rai, Ramesh Prasad Adhikari, Carol M. Worthman, Brandon A. Kohrt

**Affiliations:** 1 The Department of Psychiatry & Behavioral Sciences, The George Washington University, Washington, DC, USA; 2 Psychiatric Consultation-Liaison Service, Legacy Health, Portland, OR, USA; 3 Transcultural Psychosocial Organization (TPO) Nepal, Kathmandu, Nepal; 4 Department of Anthropology, Emory University, Atlanta, USA

**Keywords:** Armed conflict, child soldiers, interpersonal violence, sexual violence, suicide

## Abstract

**Background.:**

Suicide risk reduction is crucial for 15–29-year-old youth, who account for 46% of suicide deaths in low- and middle-income countries. Suicide predictors in high-resource settings, specifically depression, do not adequately predict suicidality in these settings. We explored if interpersonal violence (IPV) was associated with suicidality, independent of depression, in Nepal.

**Methods.:**

A longitudinal cohort of child soldiers and matched civilian children, enrolled in 2007 after the People's War in Nepal, were re-interviewed in 2012. The Depression Self-Rating Scale and Composite International Diagnostic Interview assessed depression and suicidality, respectively. Non-verbal response cards were used to capture experiences of sexual and physical IPV.

**Results.:**

One of five participants (19%) reported any lifetime suicidal ideation, which was associated with sexual IPV, female gender, former child soldier status and lack of support from teachers. Among young men, the relationship between sexual IPV and suicidality was explained by depression, and teacher support reduced suicidality. Among young women, sexual IPV was associated with suicidality, independent of depression; child soldier status increased suicidality, and teacher support decreased suicidality. Suicide plans were associated with sexual IPV but not with depression. One of 11 female former child soldiers (9%) had attempted suicide.

**Conclusion.:**

Sexual IPV is associated with suicidal ideation and plans among conflict-affected young women, independent of depression. Reducing suicide risk among women should include screening, care, and prevention programs for sexual IPV. Programs involving teachers may be particularly impactful for reducing suicidality among IPV survivors.

## BACKGROUND

Suicide accounted for 804 000 deaths worldwide in 2012 and was the second leading cause of death globally among young adults aged 15–29 years old (WHO, 2014). These youth and young adult suicides constitute 46% of suicides in low- and middle-income countries (LMIC), with the greatest youth suicide burden in Asian countries (WHO, 2014). These statistics likely underestimate incidence due to inaccurate and absent reporting and monitoring infrastructure (Hagaman *et al.* 2016; Jordans *et al.* 2014). Although psychiatric disorders remain the strongest predictor of suicidality, 31–57% of suicide attempts are not linked to prior psychiatric disorders (Nock *et al.* 2009). Indeed, such disorders poorly predict suicidality in LMIC. The relationship between mood disorders and suicidal behavior, in particular, is weaker in LMIC compared with high-income countries (HIC) (Nock *et al.* 2008). Although some studies have found a link between mental health conditions and suicide in LMIC, this association may be weaker in the South and East Asia where depression is an especially poor predictor of suicide among women (Maselko & Patel, 2008; Zhang *et al.* 2010).

Interpersonal violence (IPV) may better predict suicidality in LMIC settings, especially among women. IPV, defined as the intentional use of physical force or power against another, has a high likelihood of resulting in injury, death and psychological harm (WHO, 2013). IPV is a major public health problem in both LMIC and HIC. In the USA, two clear pathways between IPV and suicidal ideation for African-American women have been elucidated: in one pathway, IPV leads to depressive symptoms, which result in suicidal ideation; in a second pathway, IPV leads to posttraumatic stress disorder (PTSD), which results in depression and, ultimately, suicidal ideation (Leiner *et al.* 2008). A similar pathway has not been identified in LMIC, and the lack of association between depression and suicide in some LMIC populations suggests IPV may be associated directly with suicidality, independent of depression.

IPV is a product of gender norms and power relations (Jaycox *et al.* 2002; Antai & Anthony, 2014). The global lifetime prevalence of IPV among ever-partnered women is 30%, with South and East Asia regions having the highest prevalence at 37.7% (WHO, 2013). Along with gender and geography, violence, generally, and armed conflict, specifically, predict increased IPV risk. Physical and sexual violence are used as tools of war, and both women and men are at increased risk of IPV in settings of conflict and post-conflict, including camps for refugees and internally displaced persons (Turshen, 2000; Sivakumaran, 2007; Hossain *et al.* 2014). Among former child soldiers in Sub-Saharan Africa, IPV is especially common due to practices of taking of girl soldiers as ‘bush wives’ or sexual slaves (Betancourt *et al.* 2010; Coulter, 2015).

The short-term health effects of IPV, such as physical injuries, are well documented. However, researchers continue to investigate its long-term consequences. Using a cross-sectional survey of 1152 women who experienced IPV, Coker and colleagues (2002) identified that IPV was associated with long-term poor mental health outcomes, such as substance abuse, PTSD, depression, anxiety, and suicidal ideation. In a 2013 systematic review, any IPV experience increased the odds of incident depressive symptoms and suicide attempts among women, but the same association was not significant among men (Devries *et al.* 2013). Among adolescents, the closeness of a perpetrator has been related meaningfully to suicidal behavior, with violence within a dating or peer relationship having the strongest association with any suicide attempt in high school students in the USA (Swahn *et al.* 2008).

Using data from Nepal, we examined if sexual and physical IPV were associated with suicidality, either mediated by or independent of depression, and explored the potential effect of gender and exposure to armed conflict as a child soldier on this association. In the 2011 Nepal Demographic and Health Survey, 22% of reproductive-aged women reported experiencing physical violence and 12% reported experiencing sexual violence (Nepal Ministry of Health and Population, 2011). A population-based study in Nepal that included both urban and rural areas found that 16.3% of women who experienced any IPV reported a change in their mental health after the incident (Kumar *et al.* 2012). A recent psychological autopsy study of police-reported suicides in Nepal found that at least 61% of the women who died by suicide had a relative who knew they had been physically abused as an adult (Hagaman *et al.* 2017). Moreover, this study found that in rural Nepal the vast majority of these female suicides (85%) occurred before 26 years of age. However, information about the relationship among IPV, affective symptoms and suicidality remain limited in this context. In addition, few studies have investigated IPV, suicidality, and depression among persons who experienced prolonged political violence during childhood. Therefore, in settings such as Nepal, which experienced a 10-year civil war from 1996 to 2006, it is crucial to broaden the understanding of suicide predictors and then guide the development of suicide risk reduction programs.

## METHODS

### Setting

Nepal is a low-income nation, with a Human Development Index rank of 157 out of 186 countries, a life expectancy of 69.1 years, a mean of 3.2 years of education, and a gross national income per capita of US$1,137 (UNDP, 2015). At the time of the study, Nepal ranked 121st of 136 countries on the Global Gender Gap Index, a composite measure of female to male attainment (World Economic Forum, 2013). Recent legislation was designed to protect women by raising the legal age of marriage, improving inheritance rights, and prohibiting marital rape and sexual harassment, yet these laws are poorly understood and inconsistently enforced (Tuladhar *et al.* 2013).

The Nepali population experienced a decade-long civil war (1996–2006) between the People's Army of the Communist Party of Nepal (Maoist) and the government of Nepal. One of the consequences of the war was the widespread recruitment of child soldiers (Human Rights Watch, 2007), who experienced high rates of PTSD, depression, and impairments in daily functioning (Kohrt *et al.* 2008).

### Participants

Participants were part of a 5-year longitudinal study that began in 2007, a year after peace accords ended the decade-long People's War in Nepal (Kohrt *et al.* 2015*a*, *b*). The current cohort began in 2007 when we employed criterion sampling to select child soldiers and matched civilian children from eight districts across Nepal. Child soldier status was determined based on name lists of ‘Children Associated with Armed Forces and Armed Groups’ (CAAFAG) established by human rights organizations working with UNICEF (UNICEF, 2007). Consistent with the 2007 Paris Principles, CAAFAG was operationally defined as ‘any person below 18 years of age who is or who has been recruited or used by an armed force or armed group in any capacity, including but not limited to children, boys, and girls used as fighters, cooks, porters, messengers, spies, or for sexual purposes’ (UNICEF, 2007). Both children taking a direct part in hostilities and those not taking a direct part in hostilities were included, consistent with this definition (UNICEF, 2007). In this paper, we employ the more commonly used term ‘child soldier’ to refer to CAAFAG. Matched civilian children were identified through school records. Their status as non-CAAFAG (i.e., non-child soldier) was confirmed by interviews with the children and secondary review of the human rights groups’ listings.

Utilizing these selection criteria, 258 former child soldiers, including 159 male participants and 99 female participants, and a matching group of civilian children with the same gender breakdown were selected for this study (see Supplemental File 1 for sample size determination). At study entry in 2007, the average age of participants was 15.59 years (s.d. = 1.35 years, range = 11–17 years). Child soldiers were enrolled prior to participating in nongovernmental reintegration services provided by UNICEF, and a cohort of 258 civilian children matched on demographics were enrolled in the study but did not receive intervention services. All child soldiers were given the opportunity to participate in reintegration programs through UNICEF partners, the main packages of which were education, vocational training, apprenticeship and income-generating activities (Adhikari *et al.* 2014; Kohrt *et al.* 2015*a*, *b*). After enrollment in 2007, participants were followed for 1 year then re-interviewed in 2008, at which time 222 of the former child soldiers (86% of original sample) and 234 of the matched civilians (91%) participated. For the current study in 2012, attempts were made to re-interview all participants from the original study. Re-interview attempts used geographic and contact information. Initial engagement was through mobile phone numbers. When unable to reach participants in this way, researchers visited the communities where the participants lived and gathered updated contact information from teachers or members of civil society.

### Interview procedure

Participants completed 60- to 90-minute individual interviews in which research assistants read questionnaires in Nepali to participants. The interview included culturally adapted versions of mental health assessment questionnaires, developed based on findings from a semi-structured qualitative interview with child soldiers during the first wave of the study (Kohrt *et al*. 2011) (see Supplemental File 2 for semi-structured interview).

### Measures

#### Interpersonal violence

During the third wave of data collection in 2012, an IPV questionnaire was added to the instruments used in prior waves of assessment. Due to the age of participants during earlier data collection and concerns expressed by non-governmental agencies supporting them, IPV was not assessed in 2007 and 2008. In 2012, we used non-verbal response cards with sealed envelopes to assess IPV (see Supplemental File 3 for IPV questionnaire). Participants received response cards before these questions (see Supplemental File 4 for non-verbal response card set). Researchers first repeated and elaborated upon confidentiality, which had been introduced during the consent process. Researchers then gave instructions about how to use the cards, allowed opportunities for practice, and answered participant questions. After ensuring facility with the technique, researchers read the survey questions aloud while participants marked each card. This included either circling a pictorial representation or writing a number, which required basic numeric literacy shared by participants. Upon completion, participants placed the cards envelopes, sealed these, and returned them to the interviewer. Researchers were trained to pause questioning or to replace IPV questions with innocuous queries if the interview was interrupted by other individuals. Similar methods have been used in sensitive data collection, such as pocket-chart voting in Zimbabwe (Gregson *et al.* 1998), polling boxes in India (Hanck *et al.* 2008), and a similar sealed envelope method in Uganda (Barr *et al.* 2017). The sealed envelope method was selected to allow participants to respond anonymously; this is hypothesized to facilitate disclosure by reducing social desirability bias, felt-stigma, and social risks of disclosure (Barr *et al.* 2017) while preserving the ability to ask for assistance or clarification from the interviewer.

Each participant answered up to 40 questions addressing various domains of IPV victimization and perpetration. The experiences endorsed by the participant determined the number of questions that he/she was asked. Questions about violence victimization drew on the Conflict Tactics Scale and WHO Multi-Country Study on Women's Health and Domestic Violence Against Women (García-Moreno *et al.* 2005). These questions included lifetime experiences of partner violence for ever-married men and women, including emotional violence (insult, humiliate or threaten to hurt you), moderate physical violence (shake, push, slap or throw something at you), severe physical violence (beat you up, choke or burn you or threaten you with a knife or other weapon), and sexual violence (force you to perform sexual acts you found humiliating or degrading or have sex when you did not want it). For the current analyses, we created two categorical scores: any lifetime intimate partner sexual violence (yes/no) and any lifetime intimate partner physical violence (yes/no).

#### Suicidality

Suicidality questions were adapted from the Composite International Diagnostic Interview (CIDI) suicidality module (Robins *et al.* 1988), which has been adapted for use in Nepal (Jordans *et al.* 2017). Researchers asked participants whether they had ever had thoughts, plans or attempts to kill themselves in their lifetime. Participants endorsing this question then were asked about lifetime and past 12-month thoughts, plans and attempts. Sequentially, participants who responded affirmatively to the ideation question were asked if they had made a plan to take their own life. Those who responded affirmatively to the planning question were asked if they attempted to take their own life. Data on suicidality were not collected in 2007 and 2008 due to the age of participants during earlier data collection and concerns expressed by non-governmental agencies supporting them.

#### Depression

Depression was assessed using the Depression Self Rating Scale (DSRS) for children (Birleson, 1981). The DSRS is an 18-item scale assessing depression symptoms with ‘mostly,’ ‘sometimes,’ or ‘never’ on a scale of 0–2, with some items reverse coded for scoring (instrument range = 0–36). The DSRS has been transculturally translated and validated in Nepal: area under the curve = 0.82, Cronbach *α* = 0.80, test-retest reliability *r* = 0.80, sensitivity = 0.71 and specificity = 0.81 with the cutoff score ⩾14 (Kohrt *et al.* 2011).

#### Social support

Qualitative research was used to develop social support measures for groups including family members, friends, and teachers (Kohrt *et al.* 2015*b*). Social support items included ‘is proud of me,’ ‘shows interest in my experiences,’ ‘spends time/plays with me’ and ‘allows me to share my feelings.’ Response options were 0 ‘never,’ 1 ‘sometimes’ and 2 ‘often.’ A mean score was calculated for the four items within each social domain: family, friends, and teachers.

### Protection of human subjects

The Nepali NGO, Transcultural Psychosocial Organization (TPO) Nepal, implemented the formative research and intervention from 2006 to 2009, in addition to the currently described follow-up study in 2012. Ethical review boards of the Nepal Health Research Council, Emory University, and George Washington University approved the study (see Supplemental File 5 for consent form). Data were gathered by a Nepali research team employed by TPO Nepal; these men and women had backgrounds in field research and received a month-long training on qualitative and quantitative data collection and the ethics of research with vulnerable children. Participants over 18 years of age provided consent. For participants under 18 years of age, legal guardians provided consent, and minors provided assent. The research team obtained verbal consent or assent from all participants. Participants were compensated with small donations of school supplies or household goods. Participants with suicidal ideation or other acute mental health needs were referred to TPO Nepal counselors. The phone number of the counselor was given to the participant, and the counselor was informed of a potential patient. Because IPV responses were collected confidentially and responses were sealed from researchers, all participants were provided information on IPV survivor supports. This included referral information for local Child Protection Committees, human rights groups, local representatives from the Ministry of Women and Children and for the Social Welfare Council, and local TPO Nepal-trained counselors.

### Analyses

Bivariate analyses (χ^2^ tests) were conducted to compare report of sexual IPV and physical IPV with demographic characteristics. Multivariate logistic regressions were planned for three outcomes: any suicidality, suicide plans, and suicide attempts. Interaction effects on these outcomes were evaluated for gender-by-IPV, gender-by-child soldier status and IPV-by-child soldier status. When interactions were significant, analyses were split by the moderating variable. Three-step hierarchical logistical regressions were conducted to identify variables associated with suicidality. In the first step, former child soldier status (child soldier *v.* civilian), caste, age, sexual IPV and physical IPV were entered. Caste was entered into the model because prior studies have shown strong associations with mental health outcomes among child soldiers (Kohrt *et al.* 2010*a*, *b*). We used three commonly recognized groupings: high-caste Hindu (*Brahman* and *Chhetri* caste groups), low-caste Hindu (*Dalit*, formerly known as ‘untouchable’ groups), and ethnic minority groups (*Janajati*, who are commonly Buddhist but may also include Hindu groups). In the second step, social support from families, friends, and teachers were each entered separately. In the third step, categorical depression caseness (‘case’ for DSRS score ⩾ 14; ‘noncase’ for DSRS < 14) was entered. The goal of this hierarchical regression was to determine if either sexual or physical IPV was associated with suicidality when controlling for demographics, then to determine if sexual or physical IPV remained significantly associated with suicidality after controlling for various forms of social support, and finally to see if either IPV association remained significant after controlling for depression caseness. Analyses were stratified by gender and child soldier status if interaction effects were observed. All analyses were conducted with Statistical Package for Social Sciences (SPSS) version 24 (IBM Corp., 2016).

## RESULTS

Of the original 258 former child soldiers and 258 matched civilian children enrolled in 2007, 154 of the former child soldiers (60% of original participants; age at follow-up: 16–26 years) and 136 of the matched civilians (53% of original participants; age at follow-up: 15–24 years) were traceable for interviews. Approximately half (53%) were female, 57% of which were former child soldiers.

Regarding lost to follow-up indicators, participants lost to follow-up in 2012 did not differ from participants interviewed with regard to baseline PTSD scores in 2007, although there was a trend toward lost to follow-up former child soldiers having lower baseline PTSD symptom severity [(child soldiers participating in 2012 (*n* = 154): baseline 2007 CPSS score = 18.00, 95% CI 16.42–19.58; child soldiers lost to follow-up in 2012 (*n* = 104): baseline 2007 CPSS score = 15.13, 95% CI 13.20–17.05; *p* > 0.05); (civilian children participating in 2012 (*n* = 136): baseline 2007 CPSS score = 9.42, 95% CI 8.31–10.54; civilian children lost to follow-up in 2012 (*n* = 122): baseline 2007 CPSS score = 8.30, 95% CI 7.11–9.50 ; *p* > 0.05)].

Nineteen percent (*n* = 55) of the total sample reported experience any lifetime suicidal ideation, 5.2% (*n* = 15) reported plans of suicide, and 4.1% (*n* = 12) reported a suicide attempt. Among girls, the prevalence of ideation, plans, and attempts were 29.6% (*n* = 40), 8.9% (*n* = 12) and 7.4% (*n* = 10), respectively. Among boys, prevalence was 9.7% (*n* = 15), 1.9% (*n* = 3) and 1.3% (*n* = 2). Among former child soldiers, prevalence was 24.7% (*n* = 38), 7.1% (*n* = 11) and 5.8% (*n* = 9), compared with civilians, for whom prevalence was 12.5% (*n* = 17), 2.9% (*n* = 4) and 2.2% (*n* = 3). Among female former child soldiers, the prevalence was 39.0% ideation (*n* = 30), 13.0% plans (*n* = 10) and 9.1% attempts (*n* = 7).

Within the total sample, 12% reported any physical IPV, and 16% reported any sexual IPV. Table 1 provides the breakdown of respondents’ IPV experience. Females were more likely than males to experience physical IPV, with 18% of females *v.* 7% of males reporting this (see Table 2). There was no statistical difference by gender for reported rates of sexual IPV, which was reported by 19% of females compared with 14% of males. Similarly, physical IPV was more common among former child soldiers compared with civilians, but there was no difference in sexual IPV between these groups.

**Table 1.** Respondents’ exposure to interpersonal violence

Interpersonal violence questions	Civilian children, *n* (%)	Child soldiers, *n* (%)	All female, *n* (%)	All male, *n* (%)
1. Insulted, humiliated or threatened by an intimate partner	7 (5.1)	19 (12.3)	18 (13.3)	8 (5.2)
2. Shaken, pushed, slapped or had something thrown at you by an intimate partner	8 (5.9)	14 (9.1)	16 (11.9)	6 (3.9)
3. Beaten, choked, burnt, or threatened with a knife or other weapon by an intimate partner	5 (3.7)	6 (3.9)	9 (6.7)	2 (1.3)
4. Forced to perform humiliating sexual acts by an intimate partner	4 (2.9)	8 (5.2)	9 (6.7)	3 (1.9)
5. Teased, harassed, touched or shown sexual things against your will by an intimate partner	14 (10.3)	19 (12.3)	15 (11.1)	18 (11.6)
6. Forced to give or receive oral, vaginal or anal sex by an intimate partner	0 (0)	5 (3.2)	2 (1.5)	3 (1.9)
7. Forced to give or receive oral, vaginal or anal sex in exchange for something by an intimate partner	0 (0)	2 (1.3)	2 (1.5)	0 (0)

**Table 2.** Prevalence of lifetime physical and sexual interpersonal violence *(*IPV*)* by demographics and mental health status *(*N = 290*)*

	IPV any physical, *n* (%)	χ2 (*p* value)	IPV any sexual, *n* (%)	χ2 (*p* value)
Total Sample	35 (12)		46 (16)	
Gender		7.76 (0.005)		1.34 (0.250)
Girls	24 (18)		25 (19)	
Boys	11 (7)		21 (14)	
Child soldier status		7.17 (0.007)		1.32 (0.250)
Civilian	9 (7)		18 (13)	
Former child soldier	26 (17)		28 (18)	
Marital status		44.70 (0.000)		
Never married	1 (0.6)		21 (13)	2.15 (0.142)
Ever married	34 (26)		25 (19)	
Education (School Leaving Certificate, SLC, completion status)		15.14 (0.001)		0.65 (0.723)
SLC not completed	15 (24)		8 (13)	
SLC completed	15 (14)		19 (17)	
Secondary education	5 (4)		19 (16)	
Depression (categorical DSRS ⩾14 variable)		5.69 (0.017)		12.23 (0.000)
No caseness	19 (9)		23 (20)	
Depression caseness	16 (19)		23 (28)	
Any suicidality (thoughts, plans, or attempts)		8.56 (0.003)		17.75 (0.000)
No	22 (9)		27 (11)	
Yes	13 (25)		19 (35)	
Suicide plans		0.94 (0.333)		16.64 (0.000)
No	32 (12)		38 (25)	
Yes	3 (20)		8 (73)	
Suicide attempts		0.25 (0.618)		2.86 (0.091)
No	33 (12)		42 (15)	
Yes	2 (16)		4 (33)	

DSRS, depression self-rating scale

**Fig. 1.**
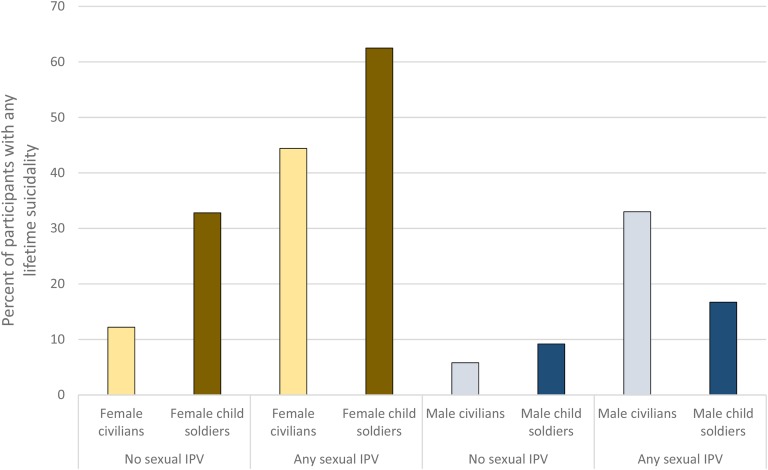
Percent of participants with any lifetime suicidality (thoughts, plans, or attempts) presented by civilian *v.* child soldier status and by exposure status for sexual interpersonal violence (IPV).

Figure 1 presents prevalence of any suicidal ideation by gender, child soldier status and exposure to sexual IPV. Multivariate logistic regressions were conducted for any suicidality, plans, and attempts, with tests of interaction effects. For any suicidality, the gender-by-child soldier status interaction was significant (*p* < 0.05), and analyses for any suicidality, therefore, were conducted for the total sample, females, and males. Hierarchical logistic regressions were conducted for the total sample, females, and males for any lifetime suicidality. For any lifetime suicidality in the total sample (Table 3a) in step one, child soldier status, sex, and sexual IPV were significant. In step two, child soldier status, sex, sexual IPV, family support and teacher support were significant, and, in step three, these remained significant along with depression. For female participants (Table 3b), child soldier status and sexual IPV were significant, and this remained in step two. In step three, teacher support was significant, in addition to child soldier status and sexual IPV, which were significant in the two prior steps. For male participants (Table 3c), there was a trend toward significance for sexual IPV in the analysis of suicidality (OR = 3.42, 95% CI 0.95–12.31). Sexual IPV and friend support were significant in step two, and teacher support and depression were significant in step three.

**Table 3.** Hierarchical multivariate logistical regression for any life time suicidality for total sample *(*2a*)*, female *(*2b*)*, male *(*2c*)*

(a) Total sample (*n* = 290; suicidality = 55, 19%)							
Total sample		Step 1		Step 2		Step 3	
		Exp. beta (95% CI)	*p* value	Exp. beta (95% CI)	*p* value	Exp. beta (95% CI)	*p* value
Child soldier		2.09 (1.05–4.14)	0.035	2.63 (1.26–5.41)	0.010	2.26 (1.06–4.83)	0.035
Caste	Janajati	Ref.	0.846	Ref.	0.476	Ref.	0.287
	Dalit	1.23 (0.53–2.84)	0.629	1.54 (0.62–3.82)	0.348	1.87 (0.73–4.77)	1.920
	Brahman/Chhetri	1.19 (0.55–2.58)	0.647	0.82 (0.35–1.90)	0.641	0.82 (0.35–1.93)	0.644
Age		0.92 (0.76–1.12)	0.440	0.92 (0.74–1.13)	0.424	0.95 (0.77–1.18)	0.696
Sex		0.27 (0.13–0.54)	0.000	0.32 (0.15–0.68)	0.003	0.36 (0.16–0.77)	0.009
IPV sexual		3.55 (1.67–7.52)	0.001	3.82 (1.68–8.69)	0.001	3.32 (1.43–7.73)	0.005
IPV physical		1.69 (0.70–4.09)	0.238	0.94 (0.36–2.44)	0.902	0.95 (0.35–2.54)	0.916
Family support		–		0.27 (0.11–0.67)	0.005	0.38 (0.15–0.99)	0.048
Friend support		–		1.25 (0.44–3.51)	0.674	1.49 (0.51–4.36)	0.460
Teacher support		–		0.42 (0.23–0.75)	0.004	0.41 (0.22–0.73)	0.003
Depression		–		–		2.91 (1.31–6.46)	0.009
(b) Female (*n* = 99; suicidality = 40, 29.6%)							
Female		Step 1		Step 2		Step 3	
		Exp. beta (95% CI)	*p* value	Exp. beta (95% CI)	*p* value	Exp. beta (95% CI)	*p* value
Child soldier		3.09 (1.27–7.57)	0.013	3.78 (1.42–10.10)	0.008	3.50 (1.30–9.43)	0.013
Caste	Janajati	Ref.	0.590	Ref.	0.414	Ref.	0.313
	Dalit	1.55 (0.48–4.98)	0.460	2.35 (0.64–8.62)	0.197	2.76 (0.72–10.49)	0.136
	Brahman/Chhetri	1.54 (0.61–3.91)	0.361	1.02 (0.27–2.83)	0.969	1.06 (0.38–2.98)	0.909
Age		0.93 (0.73–1.18)	0.530	0.96 (0.73–1.27)	0.793	1.00 (0.75–1.32)	0.986
IPV sexual		3.64 (1.39–9.56)	0.009	3.83 (1.24–11.78)	0.019	3.44 (1.09–10.80)	0.035
IPV physical		1.76 (0.63–4.97)	0.282	0.94 (0.31–2.87)	0.915	0.94 (0.30–2.90)	0.914
Family support		–		0.32 (0.11–1.02)	0.054	0.40 (0.12–1.30)	0.127
Friend support		–		0.65 (0.18–2.39)	0.514	0.76 (0.20–2.89)	0.689
Teacher support		–		0.47 (0.22–1.01)	0.054	0.46 (0.22–0.99)	0.048
Depression		–		–	–	2.07 (0.77–5.57)	0.151
(c) Male (*n* = 159, suicidality = 15, 9.7%)							
Male		Step 1		Step 2		Step 3	
		Exp. beta (95% CI)	*p* value	Exp. beta (95% CI)	*p* value	Exp. beta (95% CI)	*p* value
Child Soldier		1.13 (0.37–3.42)	0.828	1.77 (0.51–6.19)	0.367	1.14 (0.29–4.47)	0.848
Caste	Janajati	Ref.	0.879	Ref.	0.422	Ref.	0.332
	Dalit	0.89 (0.26–3.05)	0.850	0.76 (0.19–2.93)	0.687	0.95 (0.23–3.97)	0.947
	Brahman/Chhetri	0.65 (0.14–3.43)	0.614	0.29 (0.04–1.86)	0.190	0.22 (0.03–1.71)	0.146
Age		0.87 (0.63–1.21)	0.421	0.75 (0.52–1.09)	0.130	0.78 (0.053–1.14)	0.203
IPV sexual		3.42 (0.95–12.31)	0.059	4.29 (1.06–17.33)	0.040	2.89 (0.62–13.56)	0.178
IPV physical		1.56 (0.24–10.35)	0.644	1.43 (0.17–12.31)	0.744	1.44 (0.12–17.91)	0.780
Family support		–		0.11 (0.02–.62)	0.013	0.26 (0.04–1.87)	0.179
Friend support		–		8.90 (0.83–95.19)	0.070	12.46 (0.85–183.26)	0.066
Teacher support		–		0.37 (0.13–1.04)	0.059	0.29 (0.10–0.90)	0.031
Depression		–		–		8.47 (1.78–40.44)	0.007

There were no significant interaction effects for any lifetime plan of suicide in the total sample. Therefore, hierarchical regression was performed for the total sample (Table 4). Caste/ethnicity and sexual IPV remained significant through all three steps. Depression was not associated with plans. For suicide attempts, no interaction effects were significant, and none of the independent variables in the model were associated with the outcome.

**Table 4.** Hierarchical multivariate logistical regression for any life time suicide plan for total sample *(*N = 290; suicide plans = 15, 5.2%*)*

Total sample		Step 1		Step 2		Step 3	
		Exp. beta (95% CI)	*p* value	Exp. beta (95% CI)	*p* value	Exp. beta (95% CI)	*p* value
Child Soldier		2.61 (0.73–9.42)	0.142	2.71 (0.71–10.26)	0.143	2.54 (0.66–9.82)	0.177
Caste	Janajati	Ref.	0.300	Ref.	0.084	Ref.	0.075
	Dalit	2.76 (0.48–15.65)	0.252	3.41 (0.56–20.67)	0.182	3.88 (0.61–24.75)	0.151
	Brahman/Chhetri	7.01 (1.63–30.24)	0.009	5.66 (1.22–26.04)	0.026	6.10 (1.28–29.02)	0.023
Age		0.97 (0.69–1.35)	0.868	1.00 (0.69–1.46)	0.989	1.03 (0.70–1.50)	0.894
Sex		0.25 (0.06–1.03)	0.055	0.33 (0.08–1.39)	0.130	0.36 (0.09–1.50)	0.159
IPV sexual		6.21 (1.92–20.04)	0.002	5.58 (1.55–20.05)	0.008	5.19 (1.43–18.79)	0.012
IPV physical		1.16 (0.25–5.33)	0.848	0.83 (0.016–4.19)	0.824	0.85 (0.16–4.44)	0.846
Family support		–		0.37 (0.09–1.50)	0.164	0.48 (0.11–2.16)	0.341
Friend support		–		1.06 (0.20–5.58)	0.946	1.24 (0.23–6.72)	0.804
Teacher support		–		0.57 (0.19–1.73)	0.321	0.57 (0.19–1.71)	0.320
Depression		–		–		2.03 (0.46–8.96)	0.349

## DISCUSSION

We evaluated the potential role of IPV in suicidality, considered whether this relationship was mediated by or independent of depression, and explored the potential effect of gender and exposure to armed conflict as a child soldier on this association. This was motivated by studies of increased IPV and associated mental health problems in Sub-Saharan Africa, studies from South and East Asia that did not identify depression as a strong predictor for suicidality among women, and studies from LMIC populations that suggest IPV may be associated directly with suicidality, independent of depression. Three key findings emerge from our analyses:

- First, our study identified the prevalence of suicidality among persons exposed to political violence in childhood. Although both former child soldiers and civilian children potentially were exposed to armed conflict, suicidal ideation, plans, and attempts were at least twice as common among former child soldiers as civilian children. The highest prevalence was seen among female former child soldiers: 2 of 5 (40%) reported any lifetime suicidality, 1 of 7 (14%) reported making suicide plans and 1 of 11 (9%) reported attempting suicide. Given participants were 15–26 years old, this underscores that suicide is an important issue for youth in Nepal and highlights the particular vulnerability of female former child soldiers.
- Second, although physical IPV was not associated with suicidality, sexual IPV was associated with 3.6-fold greater odds of any suicidality among female participants, 3.4-fold greater odds of any suicidality among male participants and 6.2-fold greater odds of any lifetime suicide plans among all participants, when controlling for demographic variables.
- Third, sexual IPV was associated with 5.2-fold greater odds of lifetime suicide plans in the total sample, and depression was not associated with suicide plans. Among male participants, however, the association between sexual IPV and suicidality no longer was significant after controlling for depression. By contrast, sexual IPV continued to be associated with increased odds of suicidality among female participants, even when controlling for depression. This suggests depression mediates the relationship between sexual IPV and suicidality for men but not women. Taken together, these findings highlight the importance of considering IPV prevention and care as a pathway to suicide risk reduction.

Findings on an association between IPV and suicide are supported by a 10-country WHO study that reported increased emotional distress, suicidal thoughts and suicidal attempts among women reporting any IPV (Ellsberg *et al.* 2008), in addition to country-specific studies (Antai & Anthony, 2014). The current literature, however, describes complex associations among mental health, IPV, and suicidality. In a study from China, suicidality was associated with depression among men but not women (Zhang *et al.* 2010), whereas a systematic review and meta-analysis showed an association between incident depressive symptoms and suicide for women but not for men (Devries *et al.* 2013). One study from the USA suggested associations between (a) physical abuse and depression and (b) rape and depression differ by ethnicity; White women report depression more commonly following rape compared with Black and Hispanic women (Lacey *et al.* 2013).

Different forms of IPV can be associated with varied mental health outcomes. Associations between (a) physical abuse and alcohol use and (b) rape and other drug use have been reported among women in the USA (Lacey *et al.* 2013). Houry *et al.* (2006) showed an association with sexual IPV and depressive symptoms, PTSD and suicidal ideation among African-American females presenting to the emergency department. Analyses of data from the National Violence Against Women Survey (NVAWS) in the USA similarly have suggested varied types of IPV may be associated differently with mental health outcomes (Basile *et al.* 2004). The diversity of these findings suggests the potential for contextually varied associations and the consequent need to consider locally variable pathways between mental health, IPV, and suicidality. In particular, our findings reinforce the need for further research on why depression does not predict suicidality for women living in Asian LMIC (Zhang *et al.* 2010).

It also is notable that support from teachers, family members, and friends was meaningfully associated with some outcomes. Teachers play a culturally important role in Nepal. They hold positions of respect within the community, which allows them to influence parental, other adults’ and students’ behavior (Kohrt *et al.* 2015*b*, Kohrt *et al.* 2010*b*). Although this respect may contribute to the amplification of their positive behaviors throughout the community, it also may cause any negative behaviors to be perceived as acceptable and spread throughout the community (c.f Kohrt 2015*b*). For example, teachers mocked former child soldiers in their classrooms, and this behavior was mimicked by students. This may explain why teacher support – or lack thereof – played such an important role in suicidality risk among the adolescents and young adults in this study.

Findings on family support and reduced suicidality risk are consistent with a US-based study demonstrating the efficacy of Attachment-Based Family Therapy (ABFT) for decreasing suicide risk (Diamond *et al.* 2010). ABFT suggests that the quality of family relationships influences depression and suicide. Strengthening parent-adolescent bonds can be protective for adolescents to reduce suicidal ideation (Diamond *et al.* 2010). Support from friends did not show the same beneficial effect as was observed for teachers and family. The reason for this association is unclear, but adolescent boys may rely on friend support to the exclusion of adult support from family and teachers. Supportive relationships with teachers and families may suggest a greater likelihood of disclosing suicidality. Unfortunately, the disclosure of suicidality is rare in Nepal. Through mixed method psychological autopsies, Hagaman *et al.* (2017) found that fewer than one-quarter of family members reported knowledge of suicidal thoughts among persons who completed suicide. This suggests the need to promote communication pathways through which persons with suicidal ideation can disclose to family, friends health workers, and teachers.

Analyses included caste because prior studies have shown strong associations with mental health outcomes among child soldiers (Kohrt *et al.* 2010*a*, *b*). *Brahman* and *Chhetri* (high caste Hindu groups) had a greater risk of suicide plans compared with *Janajati* groups. This may be related to observed differences in emotional processing and socialization of shame and anger among high caste Hindu compared with *Janajati* children in rural Nepal (Cole *et al.* 2002, 2006). Cole *et al.* (2002) found that *Brahman* children reacted to emotionally distressing vignettes with anger, whereas *Tamang* children (an ethnic group categorized as *Janajati*) reacted with shame. Moreover, the *Brahman* children – especially girls – were reluctant to display anger following shame. For *Brahman* girls in this study, expectations to inhibit displays of anger increased with age. Higher risk of suicide may relate to the need to emotionally process anger without culturally acceptable ways to display this emotion. In a sample of 215 psychiatric outpatients in the USA, anger was associated with the acquired capability of suicidality, and this was mediated by thwarted belongingness and perceived burdensomeness (Hawkins *et al.* 2014). Further research is needed to understand how these expectations operate among *Dalit* persons, who were not included in the prior studies of emotion processing and display.

Our research uniquely examines suicidality in former child soldiers and civilian youth who experienced a decade of armed conflict in childhood. Extant literature currently does not demonstrate an increased risk of IPV in armed conflict (Tol *et al.* 2013). However, available data suggest that IPV may have comparable effects on mental health in conflict and non-conflict settings. Stark *et al.* (2017) reported that girls in the Democratic Republic of Congo who experienced IPV and reported that experience as acceptable had lower hope scores. They hypothesize that girls who experience IPV and accept traditional gender norms may feel like they ‘deserve’ that violence and, in turn, feel low hope for their future. Prior studies demonstrate the feasibility of programs delivered through humanitarian interventions to reduce the adverse mental health effects of IPV (Tol *et al.* 2013). Bass *et al.* (2013) conclude cognitive processing therapy was associated with improved depression and anxiety among sexual violence survivors in the Democratic Republic of Congo. These studies highlight both the potential benefit of psychological therapies delivered by non-specialists and the need to address cultural beliefs related to the experience of sexual violence.

In many contexts, women who have experienced IPV may have few options for help-seeking, and public knowledge about available resources may be limited. In rural Vietnam, Vung *et al.* (2009) found that women who experienced IPV do not seek care unless they are suffering from severe physical injuries; the authors attribute this, in part, to the belief that IPV is a domestic matter that should not be shared with health workers or other public institutions. Similarly, in Nepal, cultural attitudes proscribe disclosure of IPV experiences, and the majority of women do not seek care or support (Puri *et al.* 2015). In a study of behavioral scenario responses to IPV among former child soldiers in Nepal, only 31% of young women endorsed seeking family support, and 23% suggested reporting IPV to the police (Kohrt & Bourey, 2016). In this study, 69% endorsed leaving a partner perpetrating IPV only after a pregnancy was endangered by IPV.

### Limitations

A primary limitation of this study is that data on suicidality and IPV were collected only during the third wave of assessment. We cannot investigate nor infer causality from these cross-sectional data. In addition, the small sample size of 290 persons likely was underpowered to identify significant risk factors for a suicide attempt. The loss to follow up between 2007 and 2012 also may confound the observed associations. As described, characteristics for participants and non-participants in 2012 did not differ on the original outcome of interest (PTSD); however, there may have been differences on unmeasured variables. In particular, as IPV and history of suicidal behavior were not assessed in 2007, we were unable to assess potential differences based on these characteristics. Generalizability may be limited by the provision of UNICEF reintegration programs and psychosocial support to former child soldiers in this study (Kohrt *et al.* 2015*a*, *b*). This may also have influenced rates of suicidality, which might have been higher without such programs. One of the strengths of the study was the use of non-verbal response to obtain IPV data. This is hypothesized to reduce the effects of stigma and social desirability on reporting, although no systematic studies of this technique have been conducted in Nepal.

In our sample, 12% reported any physical violence, and 16% reported any sexual violence. In the majority of IPV literature, physical violence is endorsed more frequently than sexual violence. It is important to note that the difference observed in our study is not statistically significant and may reflect the small sample size. There also may be characteristics particular to this sample that led to the higher endorsement of sexual violence. Our qualitative work supports high rates of sexual violence during the decade-long political violence, in which girls described sexual violence before joining the armed conflict (e.g., girls joined the Maoists to escape sexual violence in forced marriages) (Kohrt *et al.* 2010*a*, *b*). With larger sample sizes in future studies, it would be beneficial to disaggregate IPV by type, severity, and frequency of exposure.

### Implications

Given our finding that one out of 11 female former child soldiers had attempted suicide and similar findings in Nepal, i.e., 85% of female suicides in rural areas occur before the age of 26 (Hagaman *et al.* 2017), it is imperative to find accurate predictors of suicide to guide risk reduction among young women. Our findings show a significant main effect of gender, child soldier status and sexual IPV on suicidality. It is important to identify those who experienced or are at risk for experiencing IPV and to provide psychosocial support that will reduce its long-term mental health effects, including suicidality. Sexual IPV history and risk could be assessed confidentially in primary care facilities, schools, and women's civic groups in order to identify women needing supportive services.

In Nepal, dialectical behavior therapy (DBT) has been adapted and proven to be feasible and acceptable for addressing suicide risk among women with prior histories of self-harm (Ramaiya *et al.* 2017). We recommend exploring its application specifically for women who have experienced IPV. As teachers have been identified as a source of support and thus a protective factor for former child soldiers, they are an ideal intervention source for addressing IPV and suicidality among students (Morley & Kohrt, 2013; Kohrt *et al.* 2015*b*). The Nepali DBT intervention developed by Ramaiya *et al.* (2017) currently is being piloted as a classroom-based DBT suicide prevention program for adolescents in Nepal and ultimately will include teachers’ co-facilitation of the program. In the USA, teachers have delivered school-based dating abuse prevention programs that have resulted in a reduced report of physical dating violence victimization and potentially reduced the report of sexual dating violence victimization (Foshee *et al.* 2005).

Given associations among survivors of IPV of ‘thwarted belongingness’ or ‘perceived burdensomeness’ with suicide risk, interventions could also target these two domains which are part of the Interpersonal Psychological Theory of suicide (e.g., Constantino *et al.* 2005). In conflict settings, individuals may be at a greater risk for thwarted belongingness, due to forced displacement and family separation, and perceived burdensomeness, due to changes in livelihood and having to care for oneself and family, in the context of the humanitarian crisis (Van Orden *et al.* 2010). In other settings of armed conflict, an integrated psychological and advocacy group intervention was developed to reduce psychological distress among refugee women (Tol *et al.* 2017).

## CONCLUSION

In a population affected by a decade of conflict during their youth, we identified high rates of suicidality, especially among female former child soldiers. One of five adolescents and young adults in the study had suicidal ideation during their lifetimes, and one of 11 female former child soldiers had attempted suicide. Among young women, sexual IPV was significantly associated with suicidality, whereas depression was not. Our findings suggest the need to look beyond depression screening and prevention to reduce suicide risk among young women. In particular, it would be beneficial to screen for IPV and conduct prevention programs in conflict-affected populations in this and potentially other settings. Further research should evaluate how public health initiatives to reduce IPV can contribute to reducing the burden of suicide.

## Supplementary material

For supplementary material accompanying this paper visit http://dx.doi.org/10.1017/gmh.2017.31. click here to view supplementary material
