# Supplementary material for: Interpersonal violence and suicidality among former child soldiers and war-exposed civilian children in Nepal
Source: Glob Ment Health (Camb). 2018 Feb 22;5:e9. doi: 10.1017/gmh.2017.31 (PMC5827420; doi:10.1017/gmh.2017.31)
Supplement: Supplementary file 1 [file S2054425117000310sup001.zip › SupplementalFile_5_Consent Form.pdf]

नेपालमा युवाहरुमाझ आघात तथा पुर्वावस्था ग्रहण गर्न सक्ने क्षमता सम्वन्धि अध्ययन प्रश्नावली २०६९

परिचय :- मेरो नाम .... हो र म सँग मेरो साथी................................... हुनुहुन्छ । हामी काठमाडौंको बहुसास्कृतिक मनोसामाजिक सस्थां (टि. पी. ओ .) नेपाल भन्ने संस्थाबाट आएका हौं । हाम्रो सस्थाले द्वन्द्व, प्राकृतिक प्रकोप, मानव बेचबिखन तथा ओसारपसार लगायत विभिन्न जोखिमबाट प्रभावितहरु तथा उनिहरुको परिवारको मानसिक, भावनात्मक, ब्यबहारीक र सामाजिक अवस्थामा केन्द्रित रही कार्य गर्दछ । यसै सिलसिलामा अहिले हामी सशस्त्र द्धन्द्धले गर्दा बालबालिका तथा युवा युवतिहरुको मानसिक, व्यवहारीक र सामाजिक अवस्थामा पारेका प्रभावको बारेमा कुराकानी गर्न आएका छौं । यस क्रममा अहिले हामी यस अघि दुवै पटक भेटिसकेका वालवालीका तथा युवायुवतिहरुसँग नै कुराकानी गर्ने छौं ।

उदेश्य तथा प्रक्रिया :- यस कुराकानीको मूख्य उद्देश्य भनेको तपाईको मनोसामाजिक अवस्था र तपाईले भोग्नु भएको अनुभवहरु तथा विगत ५ बर्षमा तपाईको मनोसामाजिक सुस्वास्थ्यमा कसरी परिवर्तन भएको छ भन्ने अध्ययन गर्नु हो । हामी तपाईको सकारात्मक तथा नकारात्मक अनुभवहरु, तपाईको आशाको स्तर र मनोसामाजिक अवस्थाको बारेमा कुराकानी गर्न चाहन्छौं । शुरुमा हामी तपाईसगँ कुराकानी गर्नको कारण र प्रकृयाको बारेमा बताउने छौं । त्यसपछि तपाईको अनुमति लिएर प्रश्नावलि भर्ने छौं सोहि क्रममा यदि तपाईले स्विकृति दिनु भयो भने २० मिनेटको अन्तरालमा तपाईको माझि वा साहिली औंलाबाट दुई पटक केहि थोपा रगत पनि सकंलन गर्ने छौं । त्यसको लागी हामीसगँ औलामा क्लिक गर्ने सानो मेसिन छ जुन एक पटकमा एउटा प्रयोग गर्नु पर्ने हुन्छ त्यसको माध्यम बाट हामी रगत लिनेछौं । रगत अन्तराष्ट्रिय गुणस्तरको रगतको नमुना राख्न मिल्ने कागजको टुक्रामा राखेर लिनेछौं । यसरी रगत सकंलन गर्नुको उदेस्य कसरी जिनहरु (बशानुगत तत्वहरु) तनावसगँ सम्वन्धित छन् भनेर पत्ता लगाउनु हो । तपाईले दिनु भएको जानकारीहरु यस क्षेत्रमा काम गरीरहेका राष्ट्रिय तथा अन्तराष्ट्रिय सरकारी तथा गैरसरकारी सघं सस्थाहरुका लागी एकदमै महत्वपूर्ण हुनेछ किनभने तपाईको जानकारीलाई बिष्लेषण गरी त्यसको नतिजा हामी उनिहरुलाई उपलब्ध गराउनेछौं । तर हरेक समयमा तपाईको ब्यक्तिगत पहिचानलाई गोप्य राखीनेछ । यस अध्ययनको लागि लगभग २ घण्टासम्म तपाईको समय लाग्न सक्छ ।

स्वेच्छिक सहभागिता :- यस अध्ययनमा तपाईको सहभागिता पूर्ण रुपमा तपाईको स्वेच्छामा हुनेछ । तपाई यस अध्ययनमा सहभागी नभएता पनि तपाईले कुनैपनि निकायबाट कुनै पनि सहयोग पाईरहनु भएको छ भनेपनि त्यसबाट पाईरहनु भएको सहयोगमा कुनै प्रभाव पर्ने छैन । कुराकानीको अवधिमा तपाइलाई केही समस्या वा अप्ठ्यारो परेको खण्डमा तपाई कुनै पनि बेला छलफल वा कुराकानी रोक्नको लागी हामीलाई भन्न सक्नु हुनेछ । त्यतिबेला हामीले केहि समयको लागी वा सधैको लागी यो कुराकानीलाई रोक्ने छौं ।

गोपनियता :- यहाँ हामी के भन्न चाहन्छौ भने तपाई र हामी बिचको कुराकानी गोप्य रहनेछ र हामी बिचको यी कुराकानी कुनै पत्र-पत्रिका वा सञ्चार माध्यममा प्रसारित हुने छैन । यस छलफलको क्रममा आवश्यक जानकारी नछुटोस भन्ने उद्देश्यले हामी तपाइले भनेका कुराहरू लेख्नेछौं र तपाईको अनुमति लिएर आवश्यक परेको अवस्थामा रेकर्ड पनि गर्नेछौं किनभने तपाईहरुले दिनु भएको जानकारी हामीलाई बिष्लेषण गर्नको लागी एकदमै महत्वपुर्ण हुनेछ । तपाईले दिनु भएको जानकारीको प्रतिवेदन जहाँ तपाईको पहिचान खुल्ने कुनै कुरा हुदैन त्यो मात्र हामीले अरुलाई उपलब्ध गराउने छौ जसको प्रयोग यस्तै क्षेत्रमा काम गरीरहेका सरकारी तथा गैरसरकारी निकायले राम्रोको लागी प्रयोग गर्न सक्नेछ ।

जोखीम :- यस अध्ययनमा समावेश हुँदा तपाईलाई केहि असजिलो महशुस हुन सक्नेछ किनभने हामी भावनासँग सम्वन्धीत प्रश्नहरू सोध्ने छौं र तपाईबाट केहि थोपा रगत पनि लिनेछौं । यदि तपाईलाई यसले गर्दा कुनै समस्या आएमा कृपया हामीलाई भन्नु होला हामी केहि समयको लागि रोक्ने छौं । यदि तपाईलाई अध्ययनको क्रममा र सकिएपछी पनि केही नरमाईलो महशुस भएमा हामीलाई थाहा दिनु होला हामीहरु यहाँ उपलब्ध भएका संस्थालाई मनोसामाजिक सहयोगको लागि अनुरोध गर्दछौं । तर तपाईलाई के विस्वास दिलाउन चाहन्छौं भने हामीले रगत निकालेकै कारणबाट तपाईको शरीरमा कुनै पनि किसिमको हानी पुग्ने छैन र हामीले तपाईको भावनालाई चोट पुग्ने किसिमको कुनै पनि कार्य गर्ने छैनौं ।

फाईदा :- यस अध्ययनमा समावेश भए बापत तपाईहरूलाई व्यक्तिगत रूपमा फाइदा नपुग्न पनि सक्छ तर तपाईहरूबाट पाएको जानकारीहरुको बिष्लेषणबाट यस क्षेत्रमा काम गरीरहेका राष्ट्रिय तथा अन्तराष्ट्रिय सरकारी तथा गैरसरकारी सघं सस्थाहरुले तपाई हामीहरु जस्तै युवायुवति तथा बालबालिकाहरुको भावनात्मक तथा व्यवहारिक समस्यामा सहयोग गर्न सक्छन् भन्ने हामीले आशा गरेका छौं । यस अध्ययनमा समावेश भए बापत हामी तपाइहरूलाई कुनै आर्थिक सहयोग प्रदान गर्ने छैनौं केवल तपाईलाई सहभागीताको लागी स-धन्यवाद साथ सानो उपहार प्रदान गर्न सक्नेछौं ।

अनुमति र मान्यताहरु :- अध्ययन/अनुसन्धानको कारणले कुनै पनि सहभागिलाई र अनुसन्धान गर्ने ब्यक्तिलाई हानी पुग्नु हुँदैन भन्ने अर्न्तराष्ट्रिय अभिलेखमा छ र मान्यता पनि हो । त्यसैले अध्ययन/अनुसन्धान सही तरीकाबाट हुन अति नै जरुरी हुन्छ । जुन कुरा प्रति हामी एकदमै कटिवद्ध छौं भन्ने विस्वास दिलाउन चाहन्छौं । यो अनुसन्धानको लागी नेपाल स्वस्थ्य अनुसन्धान परिषद्बाट अनुमति लिईएको छ ।

अन्य जानकारीको लागि :- यदी तपाईलाई यस अनुसन्धानको बारेमा केहि सोध्न मन लागेमा वा संस्थाको बारेमा अन्य केही जान्न मन लागेमा कृपया निम्न संस्थामा सम्पर्क राख्न सक्नुहुनेछ ।

सम्पर्क गर्ने ब्यक्ति तथा संस्था : ब्रान्डन कोहर्ट प्रमुख अनुसन्धानकर्ता , सुरज कोईराला कार्यालय प्रमुख, नगेन्द्र लुईटेल अनुसन्धानकर्ता
टि पी ओ- नेपाल, बालुवाटार, काठमाडौं नेपाल, फोन नं ०१-४४३१७१७ ईमेलः- tponepal@tponepal.org.np

हामी तपाइलाई यो लेखिएको कुराको एक प्रति दिनेछौं ।

कोड नम्वर : ....

--------------------------------- -------------------- ------------------

सहभागीको नाम मिति समय

उत्तरदाताको स्थाई ठेगाना :

जिल्लाः .... गाविस/नपा................................. वडा नं...

टोलको नाम :...

यदि मैले तपाइसँग पुन भेटनुपरेमा त्यसको लागी कृपया तपाईको पुरा ठेगाना मलाई दिन सक्नुहुन्छ होला ?

जिल्ला :...................................... गा.बि.स./नगरपालिका को नाम :... वाड नं.......... टोलको नाम.. टेलिफोन नम्बर................................... ....

अविभावकको नाम :...टेलिफोन नम्बर. ....

तपाई यस अध्ययनमा सहभागि हुन चाहनुहुन्छ ?

चाहन्छु...........................१

चाहन्न...........................२

अर्न्तवार्ताको परिणाम

१. अर्न्तवार्ता सकिएको २. अर्न्तवार्ता अपुरो भएको ३. उत्तरदाताले उत्तर दिन नमानेको

४. उत्तरदाता नभेटिएको ५. अन्य (खुलाउने) ....

यदि उत्तरदाताले उत्तर दिन नमानेमा मात्र सोध्ने , के कारणले होला ?

....

....

यदि अन्तबार्ता अपुरो भएमा त्यसको कारण खुलाउनुहोला ?

....

....

यदि उत्तरदाता नभेटिएको वा अन्य (खुलाउने) भए किन, कहाँ, कस्तो अवस्था खुलाउनुहोस् ?

....

....

....
