# Supplementary material for: Interpersonal violence and suicidality among former child soldiers and war-exposed civilian children in Nepal
Source: Glob Ment Health (Camb). 2018 Feb 22;5:e9. doi: 10.1017/gmh.2017.31 (PMC5827420; doi:10.1017/gmh.2017.31)
Supplement: Supplementary file 1 [file S2054425117000310sup001.zip › SupplementalFile_1_Sample size information (1).pdf]

In matched-cohort study conducted prior to the longitudinal sample described in the current study, we found that child soldiers had a depression score on the Depression Self Rating Scale of 14.24 (SD=5.70) and civilian children had a mean of 10.74 (SD=3.51), (Kohrt et al., 2008). In this study, the district intracluster correlation coefficient was 0.08 for depression scores. We hypothesized that the intervention would be able to reduce group differences by half, (i.e., mean difference reduced by 1.75), requiring a sample size of 41.26. At that time, non-governmental organizations estimated a minimum enrollment per district of 30 child soldiers and 30 civilian children. The design effect was 5.72, requiring 236 per arm. We therefore included 8 districts with a minimum enrollment of 30 child soldiers and 30 matched children per district.
