# Supplementary material for: Interpersonal violence and suicidality among former child soldiers and war-exposed civilian children in Nepal
Source: Glob Ment Health (Camb). 2018 Feb 22;5:e9. doi: 10.1017/gmh.2017.31 (PMC5827420; doi:10.1017/gmh.2017.31)
Supplement: Supplementary file 1 [file S2054425117000310sup001.zip › SupplementalFile_2_37-item qualitative interview.pdf]

Key Informant Interview

सम्भाव्य प्रश्नहरु:

Part I: **बाल्यकालका साधारण प्रश्नहरु**

1.
बालबालिकाहरु के कारणले गर्दा वयष्कहरुसँग भिन्न हुन्छन्?
१. बालबालिकाहरु वयष्क भएको कसरी थाहा हुन्छ?
२. बालबालिकाहरु आफ्नो कियाकलाप प्रति कहिले जिम्मेवार हुन्छन्?
३. कहिले बालबालिकाहरु विवाह गर्न सक्छन्?

2.
बालबालिकाहरुले के–कस्ता कियाकलापहरु गर्न सक्दछन्?
१. (जस्तैः घरको काम, खेतको काम, अन्य बालबालिकाहरुको हेरचाह, भारी बोक्ने काम, होटेलमा काम गर्ने, यातायात सेवा आदि)
२. बालबालिकाहरु कहिले आफ्नो समुदाय र देशको राजनैतिक कार्यमा संलग्न हुन सक्छन् ?

3.
बालबालिकाको “अधिकार” के-के होलान्? कृपया बताइदिन सक्नुहुन्छ?

4.
तपाईहरुको समुदायमा बालबालिकाहरुको लागि सबैभन्दा महत्वपूर्ण परम्परागत रितीरिवाज कुन हो? (जस्तैः पहिलो महिनावारी, वर्तबन्ध, आदि)

5.
बालबालिकाहरु र मनो-सामाजिक आघातः
१. कस्तो प्रकारको घटनाले बालबालिकाहरुलाई पिडा, पिर, दुःख (आघात) पुऱ्याउँछ? (बालबालिकाहरुमा डर, चिन्ता र तर्साइको कस्तो असर हुन्छ?)
२. बालबालिकाहरुले कसरी आफ्नो पिडा, पिर, दुःख पोख्छन्?

6.
बालबालिकाहरु र झारफुक (धाँमीझाँकी मात्र नभएर कुलदेवता पनि): साधारणतया सम्बन्धि मात्र नभई), झारफुकले बालबालिकाहरुको स्वास्थ्य, भावना र बानीव्यहोरालाई कसरी मद्दत गर्दछ?

7.
तपाइको विचारमा बालबालिकाहरु स्वभाविक रुपमा हिंसक वा अहिंसक हुन्छन्? किन?
१. बालबालिकाहरु के कारणले गर्दा रिसाह/आक्रमणकारी हुन्छन्? बालबालिकाले प्राय कस्तो किसिमको हिंसा गर्छन्?

२. हिँसाले मन र दिमाग, के लाई असर गर्छ?

Part II: **द्वन्दरत समुह बालबालिकाको अनुभवका विशेष प्रश्नहरु**

8. **द्वन्दरत समुह बालबालिकाको भर्ती**?
   बालबालिकाहरु कसरी शशस्त्र समुहमा संलग्न हुन्छन्?
   १. बालबालिकाहरु किन शशस्त्र समुहमा संलग्न हुन्छन्?
   २. बालबालिकाहरु के स्वेइच्छाले जान्छन्? यदी हो/होइन भने किन?

9. **द्वन्दरत समुह बालबालिकाको अनुभवको प्रभाव**

   बालबालिकाको विचारमा किन द्वन्द हुन्छ/भयो ?
   १. बालबालिकाहरुले विभिन्न राजनैतिक पार्टीहरुको ज्ञान, विश्वास र लक्ष्यलाई कसरी लिईराखेका छन्?

10.
   द्वन्दरत समुहको अनुभवबाट बालबालिकाहरुले के-कस्तो सबल पक्ष र सिपहरु पाउँछन्?
   १. बाट बालबालिकाहरुलाई के-कस्तो सेवा प्रदान गरिन्छ?

11.
   द्वन्दरत समुहको अनुभवबाट बालबालिकाहरुलाई कस्तो किसिमले असर पार्छ? (द्वन्दरत समुहको अनुभव भएका बालबालिकाहरु आफ्नै उमेरका अरु बालबालिकाहरुसँग भिन्न कसरी हुन्छन्?)
   १. द्वन्दरत समुहको अनुभवबाट बालबालिकाहरुलाई भावनात्मक वा व्यवहारिक कठिनाई हुन्छ?
   २. द्वन्दरत समुहको अनुभवाट परिवार र साथीभाइसँगको सामाजिक संबन्धमा कस्तो असर पर्छ?
   ३. के बालबालिकाहरु द्वन्दरत समुहको अनुभवले गर्दा आफुमा "लाज" वा "आत्मग्लानी" भएको महशुश गर्छन् होला?

12.
   द्वन्दरत समुहमा भर्ती भएर बालबालिकाहरुलाई हिंसाले कसरी असर पारेको छ? (लैंगिक भिन्नता)
   १. (स्वास्थ्य, भावना, आध्यात्मिक, व्यवहारिक र अरु कारणहरु) हिंसाले बालबालिकाहरुको आत्मा र कर्मलाई कसरी असर गर्छ?
   २. हेरेको र सुनेको विभिन्न हिंसक कुराहरुले बालबालिकालाई आफु स्वयंले हिंसा गर्न कसरी असर गर्छ? बालबालिकाहरु हिंसामा पर्दा कस्तो असर पर्छ?
   ३. के द्वन्दरत समुहको बालबालिकाहरुलाई बाट यातना पाउने गरेका छन्? यदि छ भने, यो यातनाको परिणाम के हुन्छ?

13.
   के बालबालिकाहरु शशस्त्रमा रहँदा आफ्नो जिवन र व्यवहारमा उनीहरुकै आफ्नो नियन्त्रण हुन्छ?
   १. सामान्यतया, तपाई द्वन्दरत समुहको अनुभव भएका बालबालिकालाई के सम्झनु हुन्छ – पिडित वा साहसी मान्छे?

Part III. फर्काइ र पुनरागमनका प्रश्नहरुः

समुदायमा फर्काइ

14.
i. द्वन्दको अनुभव पछि बालबालिकाहरु कसरी समुदायमा फर्कन्छन्?
ii. के सरकार र माओवादी नेताले पुनरागमनको प्रक्रियामा सहयोग गर्न सक्छन्? के उनीहरुलाई अन्य कतैबाट मद्दत मिल्छ वा उनीहरु आफै फर्कन्छन् (उदाहरणको लागिः ले मद्दत गर्छ)

15. फर्किने प्रक्रियामा परिवार, समुदाय र राजनैतिक निकायको कस्तो भुमिका हुन्छ?

16. कोही बालबालिकाहरु किन आफ्नो समुदाय नफर्किने निर्णय गर्छन्? (केही बालबालिकाहरु किन फर्किदैनन् होलान् र उनीहरुलाई के हुन्छ?)

पुनरागमन प्रक्रियामा द्वन्दरत समुहमा संलग्न बालबालिकाको चुनौतीः

17. पुनरागमन प्रक्रियामा बालबालिकाहरुले कस्तो कठिनाई पाउँछन्? विद्यालयमा, परिवारमा, काम पाउनुमा र विवाह गर्दा]

18. पुनरागमन प्रक्रियामा द्वन्दरत समुहका संलग्न महिलाले मुख्य के- कस्तो समस्याहरु भोग्छन् होला?

19. द्वन्दरत समुहका बालबालिकाको फर्काईबाट अरु स्थानिय बालबालिकाहरुलाई कस्तो असर पर्छ?

20. द्वन्दरत समुहका अनुभव लिएको बालबालिका प्रति समुदायले कस्तो प्रतिकिया देखाउँछन्?

21. तपाईलाई द्वन्दरत समुहका बालबालिकाहरु पिडित वा साहसी मान्छे, कस्तो जस्तो लाग्छ?

22. के समुदायमा यस्ता मानिसहरु छन् जसले द्वन्दरत समुहका बालबालिकाहरु सँग बदला लिन चाहन्छन्? (Probe: के समुदायको कुनै सदस्यले हिंसाको कारण द्वन्दरत समुहका बालबालिकाहरुलाई दोष दिन्छन् र उनीहरुलाई मार्न, पिट्न वा सजाय दिन चाहन्छन्?)

धाँमीभ्फाँक्री/भ्फारफुक/स्वागत समारोह

23.
बालबालिकाहरुले पुन समुदायमा फर्किदा कुनै विशेष स्वागत या सत्कार पाउँछन्?

24.
के परिवार वा समुदायले बालबालिकाको लागि कुनै रितीरिवाज/पूजाआजा गर्छन्? जस्तैः कुल देवताको पूजा, आदि)

25. बालबालिका पुनः समुदायमा फर्किदा स्थानिय धाँमीभ्फाँक्रीबाट सहयोग पाउन सक्छन्?

26. बालबालिकाहरु पुनः समुदायमा फर्किंदा धार्मिक नेताहरुले सहयोग गर्छन्?

27. द्वन्दरत समुहका बालबालिकाहरुले कुनै विशेष भावनात्मक वा आध्यात्मिक पिडा भोगेका छन्? (जस्तैः सातो जानु, भूत लाग्नु, आदी)

28. परम्परागत रितिरिवाज र झारफुक प्रती माओवादीको कस्तो धारणा छ? (माओवादीले कुनै रितीरिवाज वा परम्परागत रिति केही सिक्ने/प्रयोग गर्ने गर्दछन्?)

Part IV: उदाहरणहरु/घटनाहरु (द्वन्दरत समुहका बालबालिकाहरु सम्बन्धित)

29.
तपाई कुनै एक बालबालिकाको उदाहरण दिन सक्नुहुन्छ, जो राम्ररी समुदायमा फर्की पुनः पहिलेको जस्तै घुलमिल भएर बसेका छन् र जस्को भावना र व्यवहार राम्रो छ?

30.
तपाई कुनै एक बालबालिकाको उदाहरण दिन सक्नुहुन्छ, जसले समुदायमा पछी भावनात्मक र व्यवहारिक समस्या भोग्नु परेको थियो?

Part V: क्ष्ककगभक महिलाहरुको विषय

31. मा संलग्न भएका महिलाहरुले के-कस्तो अनुभवहरु गरेका हुन्छन्?

32.
तपाईंको विचारमा द्वन्दरत समुहमा संलग्न महिलाहरुलाई समुदायले कसरी हेर्ने गर्छन् होला?

33.
फर्किएका द्वन्दरत समुहमा संलग्न महिलाहरुले विवाह गर्न कत्तिको सम्भावना हुन्छ?

34.
द्वन्दरत समुहको अनुभव लिएका महिलाहरु यौन शोषित भएको पाइन्छन्?

Part VI. मनोसामाजिक सहयोग प्रश्नहरु

35.
कहाँवाट हरुले भावनात्मक र व्यवहारिक सहायता पाउँछन् र समुदायमा सहयोग पुऱ्याउन सक्छन्? (जस्तैः बाल क्लब, युवा क्लब, आमा समुह, आदि)

36.
यस समुदायमा विभिन्न बालबालिकाहरुलाई के कुनै विशेष सहयोगहरु उपलब्ध गराइन्छ?
(जस्तै दलित, महिलाहरु, तल्लो जनजातिहरु, आदि)

37.
तपाईको विचारमा, अनुभव भएका बालबालिकाहरुलाई समुदायमा फर्काउन अरु के- कस्तो मद्दत गर्न सकिन्छ?
