# Supplementary material for: Interpersonal violence and suicidality among former child soldiers and war-exposed civilian children in Nepal
Source: Glob Ment Health (Camb). 2018 Feb 22;5:e9. doi: 10.1017/gmh.2017.31 (PMC5827420; doi:10.1017/gmh.2017.31)
Supplement: Supplementary file 1 [file S2054425117000310sup001.zip › SupplementalFile_3_IPV Non-verbal response_questionnaire.pdf]

अमौखिक प्रतिक्रिया

तल दिइएका प्रश्नहरु कुनै पनि व्यक्तिको जीवनमा घटेको/भोगेको अप्ठ्यारा घटनाहरुको बारेमा सम्बन्धित छन् । तपाईले दिनुभएको साँचो उत्तरहरुले हामीलाई व्यक्तिहरुको भोगाईको बारेमा बुझ्न सहयोग गर्नुको साथै यस्तै घटना भोगेका व्यक्तिहरुलाई सहयोग पुऱ्याउनको लागि उपायहरु विकास गर्नमा मद्दत गर्दछ । यी कुराहरु एकदमै व्यक्तिगत भएको कारणले गर्दा हामी विशेष किसिमको कार्डहरु प्रयोग गर्नेछौं । यी कार्डहरु मार्फत् तपाईले उत्तर दिनसक्नुहुन्छ र तपाईको उत्तर गोप्य राखिनेछ: कसैले पनि तपाईलाई कस्तो किसिमको प्रश्नहरु सोधिएको थियो भनेर थाहा पाउनेछैनन् र मैले पनि तपाईको उत्तर के थियो भनेर थाहा पाउन सक्दिन । तपाईले ईमान्दारीपूर्वक सत्य उत्तर दिनु निकै आवश्यक छ किनभने नेपालमा भएका तपाईजस्तै अरु दाजुभाई तथा दिदीबहिनीलाई कसरी सहयोग गर्न सकिन्छ भन्ने कुरा हामी तपाईको उत्तरहरुद्वारा थाहा गर्न सक्छौं । तपाईले यो कुरा पनि बुझ्न आवश्यक छ कि मैले पनि तपाईको उत्तरहरु थाहा पाउनेछैन किनभने यी घटना अनुभव गरेका व्यक्तिहरुलाई कस्तो सहयोगको आवश्यक पर्छ भन्ने कुरामा म तालिमप्राप्त छैन । TPO Nepal नामक संस्थामा तालिमप्राप्त मनोविमर्शकर्ताहरु कार्यरत हुनुहुन्छ र यस संस्थाको पूर्ण जानकारी भएको कार्ड म तपाईलाई दिनेछु र तपाईले उहाँहरुलाई सम्पर्क गर्नसक्नुहुन्छ ।

उत्तरको लागि हामी तपाईलाई कार्डहरु दिनेछौं र तपाईले आफ्नो उत्तर पढेर सुनाउनुपर्नेछैन । यी कार्डहरुमा नक्साहरु छन्, त्यसैले उत्तर दिनको लागि तपाईले आफ्नो उत्तर पढेर सुनाउन कुनै जरुरी छैन । कुनै प्रश्नहरुको उत्तरको लागि तपाईले संख्या दिनुपर्ने हुन्छ ।

यी कार्डहरु कसरी प्रयोग गर्ने भन्ने बारेमा म तपाईलाई बताउनेछु र हामी दुईवटा प्रश्नहरुमा यो विधि अभ्यास गर्नेछौं । तपाईको छेऊमा मैले यी कार्डहरुको थाक राखेको छु अनि यहाँ भएको क्लिपबोर्डलाई यसरी समाउनुहोस् । *सहभागिलाई देखाउनुहोस्* । यसरी यो क्लिपबोर्ड समाएमा तपाईले आफ्नो उत्तरमा चिन्ह लगाउनसक्नुहुनेछ जुन चाँहि मैले देख्नेछैन । हरेक पटक हामी एउटा कार्डमा काम गर्नेछौं । कृपया, पहिलो कार्ड लिनुहोला र क्लिपबोर्डमा राखी उत्तर लेख्नुहोला । अभ्यासको लागि हामी अहिले यस कार्ड प्रयोग गर्नेछौं ।

तपाईले जस्तै मैले पनि त्यहि कार्ड समाईरहेको छु । दुवै कार्डहरुमा ४वटा संख्या भएका बाक्साहरु छन् । हरेक प्रश्नको लागि विभिन्न बाक्स प्रयोग गरिनेछ । पहिलो प्रश्नको लागि पहिलो बाक्स हो (औंलाले देखाउनुहोस्) दोस्रो प्रश्नको लागि यो दोस्रो बाक्स हो (औंलाले देखाउनुहोस्), तेस्रो बाक्सको लागि तेस्रो बाक्स (औंलाले देखाउनुहोस्) र त्यसैगरि चौथो प्रश्नको लागि चौथो बाक्स (औंलाले देखाउनुहोस्) हो । यो कस्तो क्रममा रहेको छ भनेर तपाईले कण्ठ गरिराख्नुपर्ने छैन । मैले प्रश्न सोध्ने बेला सहि बाक्स औंलाले देखाउनेछु ।

यो कार्ड प्रयोग गरि हरेक प्रश्नमा रहेका उत्तरहरुको अर्थ म तपाईलाई बताईदिनेछु । म तपाईलाई चर्को स्वरमा प्रश्न सोध्नेछु अनि हरेक चिन्हको बारेमा वर्णन गर्नेछु । त्यस प्रश्नको बारेमा ध्यान दिएर सोच्नुहोस् अनि कुन उत्तर दिने

हो, निर्णय गर्नुहोस् । तपाईको उत्तर ठूलो स्वरमा पढ्नुको साट्टो, कार्डमा भएको कुनै एउटा चिन्ह जसले तपाईको उत्तरलाई जनाँउछ, त्यसमा गोलो चिन्ह लगाउनुहोस् ।

आउनुहोस् अब हामी केहि प्रश्नमा अभ्यास गर्नेछौं । पहिलो बाक्सदेखि हामी शुरु गरौं । *बाक्सलाई औंलाले देखाउनुहोस् ।* पहिलो प्रश्न यस्तो रहेको छः “आज तपाईले खाना खानुभयो?” प्रश्नको बारेमा सोच्नुहोस् र निर्णय गर्नुहोस् । यदि तपाईको उत्तर “छैन” हो भने पहिलो चिन्हमा गोलो आकार कोर्नुहोस् (*औंलाले देखाउनुहोस्) :* गोलो आकारभित्र धर्का कोर्नुहोस् । यदि तपाईको उत्तर “छु” हो भने, दोस्रो चिन्हमा गोलाकार कोर्नुहोस्ः कोठेघर भएको गोलाकार (*औंलाले देखाउनुहोस्)* ।

तपाईले उत्तरमा गोलाकार कोर्नुभयो? यो अभ्यास प्रश्न भएको हुनाले, म तपाईको उत्तर हेर्नेछु । *सहभागिको उत्तर हेर्नुहोस् ।* तपाईले (पहिलो/दोस्रो चिन्ह)मा गोलाकार लगाउनुभएको रहेछ । यसको अर्थ (आज तपाईले खाना खानुभएको छ/ आज तपाईले खाना खानुभएको छैन) । के यो सहि हो?

अब हामी फेरि अर्को प्रश्नमा एक पटक अभ्यास गर्नेछौं । यस प्रश्नको लागि तपाईले दोस्रो बाक्समा रहेको चिन्हमा गोलाकार कोर्नुपर्नेछ । *बाक्सलाई संकेत गर्नुहोस् ।* “तपाई कतिको चिया पिउनुहुन्छ?” यो भनेको कदापि पिउदिन (गोलाकार भित्र धर्का तानेर*देखाउनुहोस्):* यो भनेको कहिलेकांहि हो (छोटो बारलाई संकेत गर्नुहोस्)ः यो भनेको धेरैजसो हो -लामो बारलाई संकेत गर्नुहोस्)। यो अभ्यास प्रश्न भएको हुनाले, म फेरि तपाईको उत्तर हेर्नेछु। *सहभागिको उत्तर हेर्नुहोस् ।* तपाईले *(गोलाकार भित्र धर्का भएको छोटो वा लामो बार)*मा गोलाकार लगाउनुभएको रहेछ । यसको अर्थ तपाईले (कदापि, कहिलेकाहि, वा धेरैजसो) चिया पिउनुहुन्छ । के यो सहि हो?

जब म कार्डमा भएका सबै प्रश्नहरु सोधिसक्नेछु तब तपाईले त्यस कार्डलाई घोप्टो पारी तपाईको शरिरको पल्लो छेऊमा राख्नुहोस् । अभ्यासका लागि केवल दुई प्रश्नहरु मत्र छन् र मलाई विश्वास छ तपाईले गर्नसक्नुहुन्छ भनेर । हामी अब नयाँ कार्डबाट प्रश्नहरु सोध्नेछु । *आवश्यक परेमा सहभागिलाई सहयोग गर्नुहोस् ।*

जब सबै प्रश्नहरु सोधिसक्नुहुनेछ, सबै कार्डहरु एउटा खाम भित्र हाली त्यस खाममा मोहर लगाउनुहोस् । तपाईको नामलाई दिईएको संख्या म यस खाममाथि लेख्नेछु । अर्न्तवार्ता पश्चात् म यस खाम नता खोल्नेछु न ती कार्डहरुलाई म हेर्नेछु । अरु कुनै व्यक्ति जसले तपाई र तपाईको नाम चिन्नुहुदैंन, त्यस्ता व्यक्तिले यस जानकारी कम्प्यूटरमा राख्नुहुनेछ ।

तपाईलाई केहि प्रश्न भएमा जति खेर पनि सोध्नसक्नुहुनेछ । म फेरि पनि भन्छु कृपया तपाईको उत्तर मलाई नदेखाउनुहोस् र त्यसलाई नपढिदिनुहोस् । याद राख्नुहोस् मैले तपाईको उत्तर थाहा पाउनुहुदैंन । शुरु गर्नभन्दा अघि तपाईमा केहि प्रश्नहरु छ?

१. तपाईको (यस अघिको वा विगत्) श्रीमान्/श्रीमतीले रक्सी सेवन (गर्नुहुन्थ्यो/गर्नुहुन्छ)?
यो भनेको *गर्नुहुदैंन/गर्नुहुदैंन थियो* हो ।
यो भनेको *गर्नुहुन्छ/गर्नुहुन्थ्यो* हो ।

२. (यस अघिको) तपाईको श्रीमान्/श्रीमतीले तपाईले कहिल्यै अपमान वा चोट पुऱ्याउने धम्की (दिनुहुन्थ्यो/दिनुहुन्छ)?

यो भनेको *दिनुहुदैंन / दिनुहुदैंन थियो* हो ।
यो भनेको *दिनुहुन्छ / दिनुहुन्थ्यो* हो ।

२.क. विगत् एक वर्षमा, यस्तो कतिपटक भएको थियो?
यो भनेको *कदापि थिएन* हो ।
यो भनेको *कहिलेकाहि* हो ।
यो भनेको *धेरैजसो* हो ।

३. (यस अघिको वा विगत्) तपाईको श्रीमान् / श्रीमतीले तपाईलाई कहिल्यै धकाल्ने, पिट्ने वा तपाईमाथि केहि सामान फ्याक्ने (गर्नुहुन्थ्यो / गर्नुहुन्छ)?
यो भनेको *गर्नुहुदैंन / गर्नुहुदैंन थियो* हो ।
यो भनेको *गर्नुहुन्छ / गर्नुहुन्थ्यो* हो ।

३.क. विगत् एक वर्षमा, यस्तो कतिपटक भएको थियो?
यो भनेको *कदापि थिएन* हो ।
यो भनेको *कहिलेकाहि* हो ।
यो भनेको *धेरैजसो* हो ।

४. (यस अघिको वा विगत्) तपाईको श्रीमान् / श्रीमतीले तपाईलाई कहिल्यै हिर्काउने, घाँटी न्याक्ने वा जलाउने, वा चक्कु अथवा अरु कुनै हतियार देखाएर धम्की (दिनुहुन्थ्यो / दिनुहुन्छ)?
यो भनेको *दिनुहुदैंन / दिनुहुदैंन थियो* हो ।
यो भनेको *दिनुहुन्छ / दिनुहुन्थ्यो* हो ।

४.क. विगत् एक वर्षमा, यस्तो कतिपटक भएको थियो?
यो भनेको *कदापि थिएन* हो ।
यो भनेको *कहिलेकाहि* हो ।
यो भनेको *धेरैजसो* हो ।

५. (यस अघिको वा विगत्) तपाईको श्रीमान् / श्रीमतीले तपाईलाई कहिल्यै तपाईलाई तपाईको इच्छाविपरितयौन सम्बन्ध राख्न वा अपमानित यौन कार्य (गर्नलगाउनुभएको थियो / गर्नलगाउनुहुन्छ)?
यो भनेको *गर्नुभएको छैन / गर्नुभएको थिएन थियो* हो ।
यो भनेको *गर्नुभएको छ / गर्नुहुन्छ* हो ।

५.क. विगत् एक वर्षमा, यस्तो कतिपटक भएको थियो?
यो भनेको *कदापि थिएन* हो ।
यो भनेको *कहिलेकाहि* हो ।
यो भनेको *धेरैजसो* हो ।

६. महिलाहरुको लागि मात्रः यी माथि उल्लेखित कुनै कुराहरु तपाईले पहिलो पटक अनुभव गर्नुभएको थियो अथवा गर्भवति वा सुत्केरि अवस्थामा यो अझ बढि खस्कदैं गएको थियो?
यो भनेको *हो* हो ।
यो भनेको *होइन* हो ।

६.क. महिलाहरुको लागि मात्रः तपाईको गर्भमा भएको बच्चा छोरा थियो कि छोरी थियो कि तपाईलाई यसबारे थाहा थिएन?

यो भनेको *केटा* हो ।
यो भनेको *केटी* हो ।
यो भनेको म/मेरो श्रीमान्लाई हाम्रो बच्चाको लिङ्गबारे थाहा थिएन ।

७. *सशस्त्र समुहमा संलग्न बालबालिका* महिलाको लागि: तपाईको श्रीमान् वा ससुरालमा तपाई माओवादीमा सम्लग्न भएको कुरा थाहा छ?
यो भनेको *हो* हो ।
यो भनेको *होइन* हो ।
यो भनेको *मलाई थाहा छैन । उहाँहरुलाई थाहा हुन पनि सक्छ वा थाहा नहुन पनि सक्छ ।*

७.क. *सशस्त्र समुहमा संलग्न बालबालिका* महिलाको लागि: तपाईले यी कुराहरु पहिलो पटक अनुभव गर्नुभएको थियो कि तपाई माओवादीमा सम्लग्न भएको कुरा थाहा पाईसकेपछि अझ साह्रो भएको थियो?
यो चाँहि उनीहरुलाई थाहा *छैन वा यो पहिलो पटक भएको होइन वा अझ साह्रो हुदैं गएको हो ।*
यो भनेको *हो* हो ।
यो भनेको *होइन* हो ।

८. यी कुराहरु हुँदा, के तपाईले कहिल्यै हान्नुभयो वा झगडा गर्नुभयो?
यो भनेको *हो* हो ।
यो भनेको *होइन* हो ।
यदि यीमध्ये केहि कुराहरु पनि तपाईमाथि भएको छैन भने, कृपया *होइन* मा गोलाकार लगाउनुहोला

९. तपाईको (विगत/यस अघिको) श्रीमान् (श्रीमती) ले तपाईलाई शारीरिक रुपमा चोट नपुऱ्याउदा नपुऱ्याउदै पनि के तपाईले उहाँलाई कहिल्यै पिटेको, झाप्पड हानेको, लात्ता हानेको वा केहि शारीरिक चोट पुऱ्याउनुभएको थियो?
यो भनेको *हो* हो ।
यो भनेको *होइन* हो ।
यदि तपाईको (विगत/यस अघिको) श्रीमान् (श्रीमती) ले तपाईलाई शारीरिक रुपमा चोट पुऱ्याउनुभएको थिएन भने, कृपया *होइन* मा गोलाकार चिन्ह लगाउनुहोला ।

९. क. विगत् एक वर्षमा, यस्तो कतिपटक गर्नुभएको थियो?
यो भनेको *कदापि थिएन* हो ।
यो भनेको *कहिलेकाहि* हो ।
यो भनेको *धेरैजसो* हो ।

१०. तपाईको (विगत/यस अघिको) श्रीमान्/श्रीमतीदेखि तपाई कतिको डराएको महशुस गर्नुहुन्छ?
यो भनेको *कदापि छैन/थिएन* हो ।
यो भनेको *कहिलेकाहि* हो ।
यो भनेको *धेरैजसो* हो ।

अब तलका प्रश्नहरुले तपाईको (विगत/यस अघिको) श्रीमान्/श्रीमतीबाहेक अरु व्यक्तिहरुले गरेको क्रियाकलापहरुको बारेमा सोध्नेछ । *सहभागीहरु जसले एकपटकभन्दा बढी विवाह गर्नुभएको छ:* यदि तपाई एकपटकभन्दा बढी विवाह गर्नुभएको छ भने, तपाईको पहिलो श्रीमान्/श्रीमती वा अरु कुनै व्यक्तिले यी क्रियाहरु गर्नसकेको हुनसक्नेछ ।

तपाईको जीवनकालको कुनै अवस्था बाल्यकाल वा वयस्क समय सम्झदाखेरी:

११. तपाईको अहिलेको (विगत/यस अघिको) श्रीमान्/श्रीमतीबाहेक अरु कसैले तपाईलाई जिस्काएको, अपमान गरेको, छोएको वा तपाईले नचाहदा नचहादैं पनि गुप्ताङ्ग देखाएको छ?

यो भनेको *छ* हो ।

यो भनेको *छैन* हो ।

१२. तपाईको अहिलेको (विगत/यस अघिको) श्रीमान्/श्रीमतीबाहेक अरु कसैले तपाईको इच्छाविपरितमौखिक, गुप्ताङ्ग यौन क्रिया लिन जबरजस्ती गरेको थियो?

यो भनेको *गरेको थियो* हो ।

यो भनेको *गरेको थिएन* हो ।

१३. तपाईको अहिलेको (विगत/यस अघिको) श्रीमान्/श्रीमतीबाहेक अरु कसैले तपाईलाई पैसा, काम, परिवारको सुरक्षा अथवा अरु केहि कुराको लोभ लालसा देखाएर तपाईलाई मौखिक, गुप्ताङ्ग यौन क्रिया गर्न (लिन वा दिन) जबरजस्ती गरेको थियो?

यो भनेको *गरेको थियो* हो ।

यो भनेको *गरेको थिएन* हो ।

१४. यी मध्ये कुनै घटनाहरु तपाई घरदेखि टाढा रहंदा, नेपालभित्र वा बाहिर हुँदा घटेको थियो?

यो भनेको *घटेको थियो* हो ।

यो भनेको *घटेको थिएन* हो ।

१५. यस्ता क्रियाहरु तपाईको जीवनमा तपाईको श्रीमान्/श्रीमतीबाहेक अरु व्यक्तिले कतिपटक जति गरेको थियो?

यो भनेको *कदापि थिएन* हो ।

यो भनेको *एक पटक* हो ।

यो भनेको *दुई पटक* हो ।

यो भनेको *तीन वा सोभन्दा बढी* हो ।

१५.क. यस्तो घटना तपाईको जीवनमा पहिलो पटक तपाई कति उमेरको हुँदा घटेको थियो?

कृपया दिएको धर्कामा तपाईको उमेर लेख्नुहोला ।

१५.ख. यस्तो घटना तपाईको जीवनमा अन्तिम पटक तपाई कति उमेरको हुँदा घटेको थियो?

कृपया दिएको धर्कामा तपाईको उमेर लेख्नुहोला ।

१५.ग. *सशस्त्र समुहमा संलग्न बालबालिका* हरुका लागि, यो घटना कहिले घटेको थियो?

यो जतिपटक भएको थियो ती सबै पलहरुमा गोलाकार कोर्नुहोला ।

यो भनेको *माओवादीमा सम्लग्न हुन अघि* हो ।

यो भनेको *माओवादीमा सम्लग्न हुदाँखेरि* हो ।

यो भनेको *माओवादीमा सम्लग्न भएपछि* हो ।

१६. तपाईले कसैलाई पिट्न वा हिर्काउनभएको छ?

यो भनेको *छ* हो ।

यो भनेको *छैन* हो ।

१६.क. तपाईले कतिपटक अरु व्यक्तिहरुलाई पिट्न वा हिर्काउनभएको छ?

यो भनेको *कदापि छैन* हो ।

यो भनेको *एक पटक* हो ।

यो भनेको *दुई पटक* हो ।
यो भनेको *तीन वा सोभन्दा बढी* हो ।

१६.ख. तपाई कति उमेरको हुनुहुन्थ्यो?
कृपया तपाईको उमेर दिएको धर्कामा लेखिदिनुहोला ।

१६.ग. यस कार्य तपाईको इच्छाविपरित कसैले जबरजस्ती गर्नलगाएको थियो?
यो भनेको *हो* हो ।
यो भनेको *होइन* हो ।

१७. तपाईले कुनै व्यक्तिलाई यातना दिनुभएको छ?
यो भनेको *छ* हो ।
यो भनेको *छैन* हो ।

१७.क. तपाईले कुनै व्यक्तिलाई कति पटक यातना दिनुभएको छ?
यो भनेको *कदापि छैन* हो ।
यो भनेको *एक पटक* हो ।
यो भनेको *दुई पटक* हो ।
यो भनेको *तीन वा सोभन्दा बढी* हो ।

१७.ख. तपाई कति उमेरको हुनुहुन्थ्यो?
कृपया तपाईको उमेर दिएको धर्कामा लेखिदिनुहोला ।

१७.ग. यस कार्य तपाईको इच्छाविपरित कसैले जबरजस्ती गर्नलगाएको थियो?
यो भनेको *हो* हो ।
यो भनेको *होइन* हो ।

१८. तपाईले कुनै व्यक्तिको हत्या गर्न कोशिस गर्नुभएको छ?
यो भनेको *छ* हो ।
यो भनेको *छैन* हो ।

१८.क. तपाईले कुनै व्यक्तिको कति पटक हत्या गर्न कोशिस गर्नुभएको छ?
यो भनेको *कदापि छैन* हो ।
यो भनेको *एक पटक* हो ।
यो भनेको *दुई पटक* हो ।
यो भनेको *तीन वा सोभन्दा बढी* हो ।

१८.ख. तपाई कति उमेरको हुनुहुन्थ्यो?
कृपया तपाईको उमेर दिएको धर्कामा लेखिदिनुहोला ।

१८.ग. यस कार्य तपाईको इच्छाविपरित कसैले जबरजस्ती गर्नलगाएको थियो?
यो भनेको *हो* हो ।
यो भनेको *होइन* हो ।

१९. तपाईले कुनै व्यक्तिको हत्या गर्नुभएको छ?

यो भनेको *छ* हो ।
यो भनेको *छैन* हो ।

१९.क. तपाईले कति पटक हत्या गर्नुभएको छ?
यो भनेको *कदापि छैन* हो ।
यो भनेको *एक पटक* हो ।
यो भनेको *दुई पटक* हो ।
यो भनेको *तीन वा सोभन्दा बढी* हो ।

१९.ख. तपाई कति उमेरको हुनुहुन्थ्यो?
कृपया तपाईको उमेर दिएको धर्कामा लेखिदिनुहोला ।

१९.ग. यस कार्य तपाईको इच्छाविपरित कसैले जबरजस्ती गर्नलगाएको थियो?
यो भनेको *हो* हो ।
यो भनेको *होइन* हो ।

२०. कुनै व्यक्तिको इच्छाविपरित तपाईले कहिल्यै यौन सम्पर्क राख्नुभएको छ?
यो भनेको *छ* हो ।
यो भनेको *छैन* हो ।

२०.क. कुनै व्यक्तिको इच्छाविपरित तपाईले कतिपटक यौन सम्पर्क राख्नुभएको छ?
यो भनेको *कदापि छैन* हो ।
यो भनेको *एक पटक* हो ।
यो भनेको *दुई पटक* हो ।
यो भनेको *तीन वा सोभन्दा बढी* हो ।

२०.ख. तपाई कति उमेरको हुनुहुन्थ्यो?
कृपया तपाईको उमेर दिएको धर्कामा लेखिदिनुहोला ।

२०.ग. यस कार्य तपाईको इच्छाविपरित कसैले जबरजस्ती गर्नलगाएको थियो?
यो भनेको *हो* हो ।
यो भनेको *होइन* हो ।

अमौखिक प्रतिक्रियाको समाप्त

२१. हामीले धेरै विषयमा कुराहरु गर्‍यौं । तपाईले यी कुराहरुको लागि कहिल्यै कसैसँग सहयोग खोज्नुभयो?
खोजेको थिए १
खोजेको थिएन २

२२. कससँग तपाईले सहयोग खोज्नुभयो? *यदि सबै लागुभएमा सबै छान्नुहोस् ।*
१. परिवारको सदस्य
२. साथी
३. महिलाहरुको संस्था
४. डाक्टर, स्वास्थ्यकर्मी, मनोविमर्शकर्ता वा मेडिकल कार्यकर्ता
५. धामी झाक्री वा धर्मगुरु
६. प्रहरी

७. अन्य व्यक्ति वा संस्था ....
