# Supplementary figures and images for: Interpersonal violence and suicidality among former child soldiers and war-exposed civilian children in Nepal
Source: Glob Ment Health (Camb). 2018 Feb 22;5:e9. doi: 10.1017/gmh.2017.31 (PMC5827420; doi:10.1017/gmh.2017.31)

१२

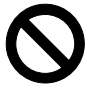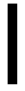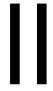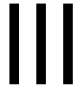

१२क

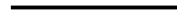

१२ख

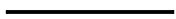

१२ग

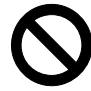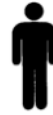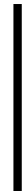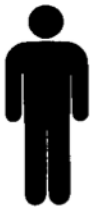

१२

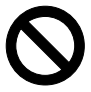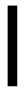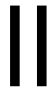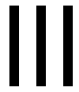

१२क

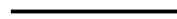

१२ख

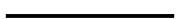

१२ग

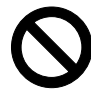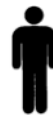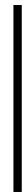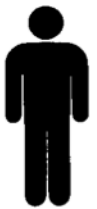

Supplement: Supplementary file 1 [file S2054425117000310sup001.zip › SupplementalFile_4_IPV Non-verbal response-CARD SET.pdf]
